# Enzymatic degradation of organophosphorus insecticides decreases toxicity in planarians and enhances survival

**DOI:** 10.1038/s41598-017-15209-8

**Published:** 2017-11-09

**Authors:** Laetitia Poirier, Lucile Brun, Pauline Jacquet, Catherine Lepolard, Nicholas Armstrong, Cédric Torre, David Daudé, Eric Ghigo, Eric Chabrière

**Affiliations:** 1 CNRS UMR 7278, IRD198, INSERM U1095, APHM, Institut Hospitalier Universitaire Méditerranée-Infection, Aix-Marseille Université, 19-21 Bd Jean Moulin, 13005 Marseille Cedex 05, France; 2 Gene&GreenTK, 19-21 Bd Jean Moulin, 13005 Marseille Cedex 05, France

## Abstract

Organophosphorus insecticides (OPs) are toxic compounds used for agricultural purposes and responsible for severe types of contamination worldwide. OPs may also induce chronic deleterious effects and developmental disruption. Finding remediation strategies is a major concern to diminish their impact on environment and human health. Enzymes have emerged as a promising eco-friendly route for decontaminating OPs. The enzyme *Sso*Pox from the archaea *Sulfolobus solfataricus* has been particularly studied, considering both its tremendous stability and phosphotriesterase activity. However, the toxicity of the degradation products generated through enzyme hydrolysis has been poorly investigated. To address both neurotoxicity and developmental perturbation, freshwater planarians from Platyhelminthes were considered to evaluate the impact of OP and degradation product exposure. Planarians have a large proportion of stem cells that give them an unconventional capacity for regeneration. OPs were found to be highly toxic to planarians and enzyme decontamination drastically enhanced survival rate. Although not completely innocuous, the degradation products were found to be less toxic than insecticides and reduced poisoning effects by increasing NOEC values by up to eight-fold. *Sso*Pox also limited detrimental consequences on planarian mobility and enabled them to recover a non-exposed type regeneration process suggesting that enzymatic decontamination is a promising alternative to bioremediation.

## Introduction

Organophosphorus pesticides (OPs) are highly toxic chemicals that have been widely developed for agricultural purposes since the 1950s^1^. Their widespread use has led to serious contamination worldwide and constitutes a major health and environmental issue^2^. The acute toxicity of OPs is due to their capacity to inhibit acetylcholinesterase, a key enzyme involved in the overall regulation of the central and peripheral nervous system. OPs disrupt nervous signals by deactivating AChE through phosphorylation and irreversible binding to the catalytic serine causing acetylcholine accumulation and cholinergic overstimulation^3^. Exposure to high doses of OPs is associated with many symptoms including salivation, diaphoresis, emesis, miosis, and tremors that may cause paralysis and death^4,5^. Furthermore, low dose chronic exposure to OPs may have deleterious effects including impaired neurobehavior performance and may negatively affect human fetal and childhood health^6,7^. Some reports have also pointed out the possible link between low level chronic exposure to OPs and health problems^8,9^.

Considering the high toxicity of OPs and their intensive use, finding efficient methods of decontamination is a major concern. Numerous approaches, including chemical, physical and biological methods, have been considered for developing decontamination strategies against OPs. However, these methods usually involve harsh conditions, are not compatible with the decontamination of people, and cannot be considered for large-scale environmental remediation^10^. Recently, enzymes have emerged as a promising alternative to smoothly and quickly decontaminate OPs. Of these, the phosphotriesterase-like lactonase *Sso*Pox from the archaea *Sulfolobus solfataricus* is an appealing candidate for remediation. This enzyme has been engineered and proven to be highly efficient for degrading a broad panel of OPs^11–14^. This enzyme displays a phosphotriesterase activity that enable to degrade OPs through an hydrolysis reaction leading to two degradation products, a phosphodiester and an alcohol. Due to its extremophilic origin, *Sso*Pox is also tremendously robust, including resistance to solvents and detergents^15^ and long-term storage or activity over a wide range of temperatures^16^. The enzyme is, moreover, highly convenient for immobilization and could be used in filtration devices to treat effluents soiled with OPs^16–18^. Recently, *Sso*Pox was engineered and variants with drastically enhanced phosphotriesterase activity were generated (manuscript in revision). Of these, variant *Sso*Pox-αsD6 was of utmost interest and it is this which we considered in this study.

Although enzyme decontamination would decrease the neurotoxicity of OPs, little is known about the toxicity of the generated degradation products and little research effort has been dedicated to their fate. This may require further consideration, as these molecules may affect the environment and non-targeted organisms^19^. The lack of a convenient and easy-to-handle *in vivo* model that could be used to assess both neurotoxicity and developmental perturbation may explain the low levels of attention that have been given this topic. Freshwater planarians, belonging to Platyhelminthes, have a large proportion of stem cells that give them an unconventional capacity for regeneration and are, subsequently, highly interesting for developmental studies^20,21^. Indeed, planarians appear to be particularly relevant for investigating developmental disruption in addition to classic toxicological investigations. Recently, planarians have emerged as an alternative animal model for toxicology and neurotoxicology^22,23^. *Dugesia japonica* ^24^, *D. gonocephala* ^25^ *, D. dorotocephala* ^26^, and *D. tigrina* ^27^ have been shown to be negatively affected by OP exposure. Cholinesterase activity was demonstrated in planarians and their sensitivity to OPs has been highlighted albeit lower than *Homo sapiens* AChE (*Hs-*AChE)^24^. Moreover, evolutionary considerations have suggested that AChE from Platyhelminthes, including planarian and *Schistosoma*, are probably early cholinesterase related to other lower vertebrates such as the medaka *Oryzias latipes* and the hagfish *Myxine glutinosa* ^28–30^. Such evolutionary trajectory may explain the difference in sensitivity to OP exposure with higher vertebrates for which a gene duplication event lead to a divergence between AChE and butyrylcholinesterase^31^.

Here, we investigated the toxicity of four widely used insecticides, paraoxon-ethyl, parathion-ethyl, diazinon and fenitrothion, which have been widely used in agriculture, as well as their respective degradation products generated by enzymatic hydrolysis with *Sso*Pox-αsD6. Mortality, behavior and regeneration were assessed in *Schmidtea mediterranea*. OPs were shown to be toxic to planarians and to affect both mobility and regeneration capacity while the use of a decontaminating enzyme drastically decreased the deleterious effects of OPs. The global toxicity of both pesticides and their corresponding degradation products were thus assessed and the results underlined the strong potential of *Sso*Pox for bioremediation.

## Results

### GC/MS analysis of enzymatically-generated degradation products

**Figure 1:**
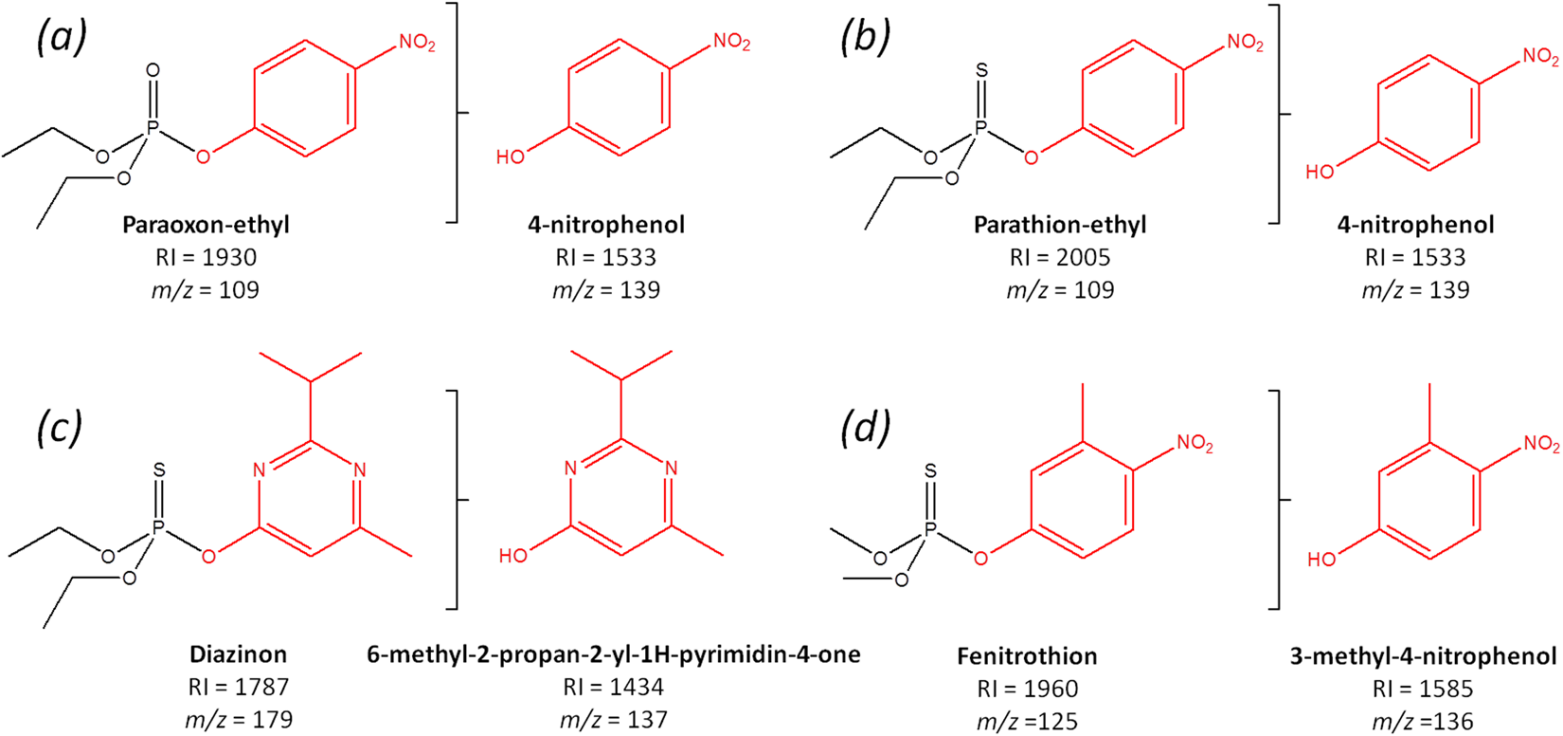
GC/MS characterization of the enzymatically-generated degradation products. RI is the experimental Kovats retention index of the molecule and *m/z* is the mass-to-charge ratio of the base peak fragment detected for each molecule. The leaving group and the corresponding degradation product are emphasized in red. (**a**) Paraoxon-ethyl (CAS 311-45-5) led to 4-nitrophenol (CAS 100-02-7); (**b**) Parathion-ethyl (CAS 56-38-2) led to 4-nitrophenol (CAS 100-02-7); (**c**) Diazinon (CAS 333-41-5) led to 6-methyl-2-propan-2-yl-1H-pyrimidin-4-one (CAS 2814-20-2); (**d**) Fenitrothion (CAS 122-14-5) led to 3-methyl-4-nitrophenol (CAS 58864-63-4).

*Sso*Pox has been previously reported to efficiently degrade a broad spectrum of organophosphorus insecticides. Here, the cleavage site for each substrate was unambiguously assigned. The leaving groups generated through enzyme hydrolysis were characterized by GC/MS and were consistent with the phosphotriesterase mechanism of *Sso*Pox. Paraoxon-ethyl and parathion-ethyl hydrolysis led to 4-nitrophenol while diazinon and fenitrothion led to 6-methyl-2-propan-2-yl-1H-pyrimidin-4-one and 3-methyl-4-nitrophenol respectively **(**Fig. 1 **)**.

### Phylogeny

**Figure 2:**
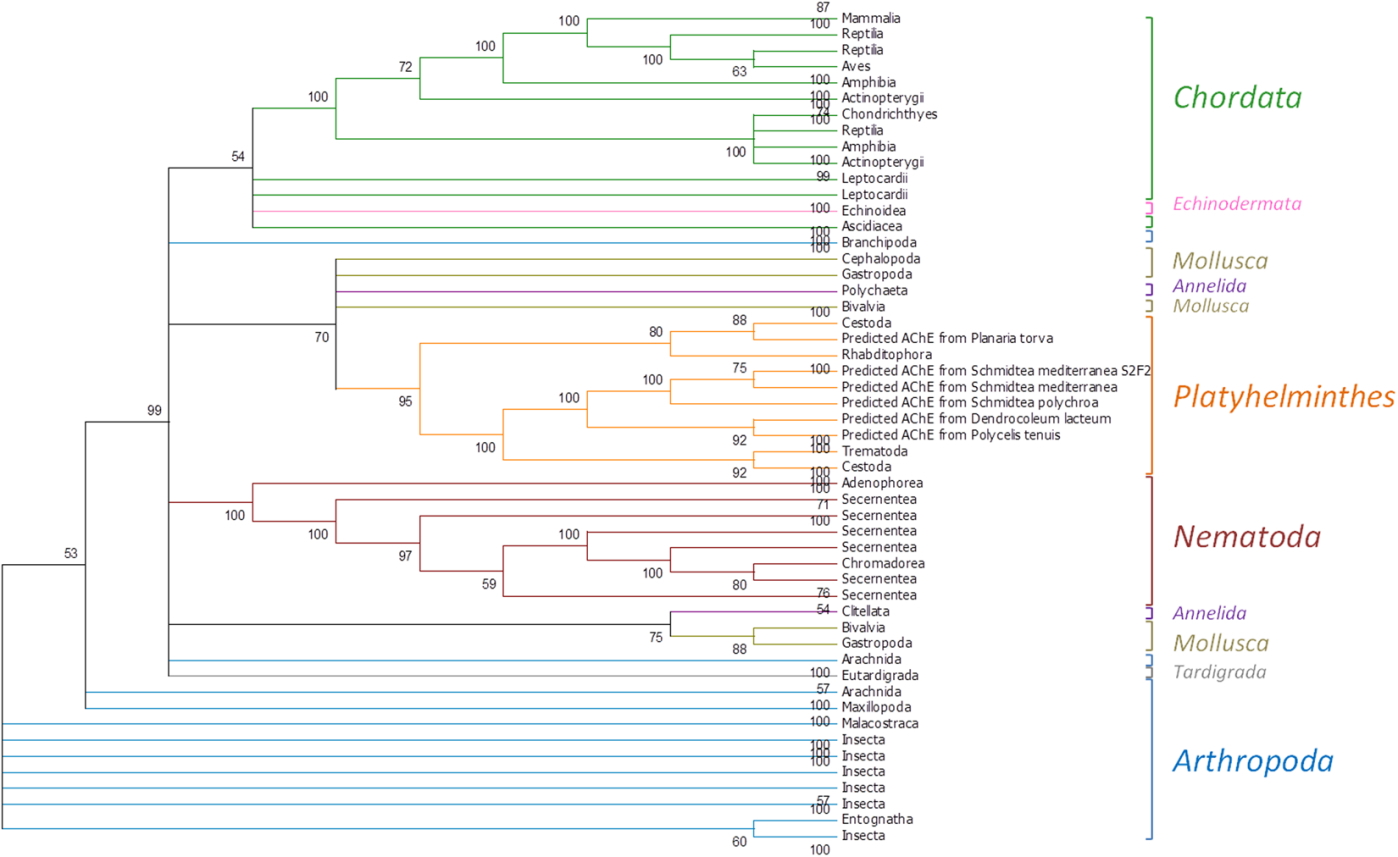
Phylogenetic tree of AChE. AChE from planarians were predicted using PlanMine (http://planmine.mpi-cbg.de)^33^ and used as template for BLAST in UNIPROT (http://www.uniprot.org/blast/). Sequence alignment and Phylogeny were performed with MEGA^34,35^. Predicted AChE from planarians were found to be closely related to other species from Platyhelminthes.

Phylogenetic analysis was performed to evaluate the evolutionary trajectory of putative planarian AChE as compared to characterized AChE from other phyla (Fig. 2). AChE sequences from *Schmidtea mediterranea*, *S. polychroa*, *Dendroceleum lacteum*, *Planaria torva* and *Polycelis tenuis* were predicted and found to be closely related to other classes belonging to Platyhelminthes. These AChE are referred to here as *Smed*-AChE, *Spol*-AChE, *Dlac*-AChE, *Ptor*-AChE, *Pten*-AChE following the gene nomenclature guidelines previously described^32^. *Smed*-AChE, *Spol*-AChE, *Dlac*-AChE and *Pten*-AChE shared more than 60% identity at the amino acid level, while *Ptor*-AChE shared only 35%. AChE from planarians also appear to share a common ancestor with Chordata and shared on average 35% identity at the amino acid level with *Hs*-AChE, suggesting that planarians may constitute a potential alternative for the study of OP poisoning.

### Acetylcholinesterase activity in Schmidtea mediterranea (Smed-AChE)

**Figure 3:**
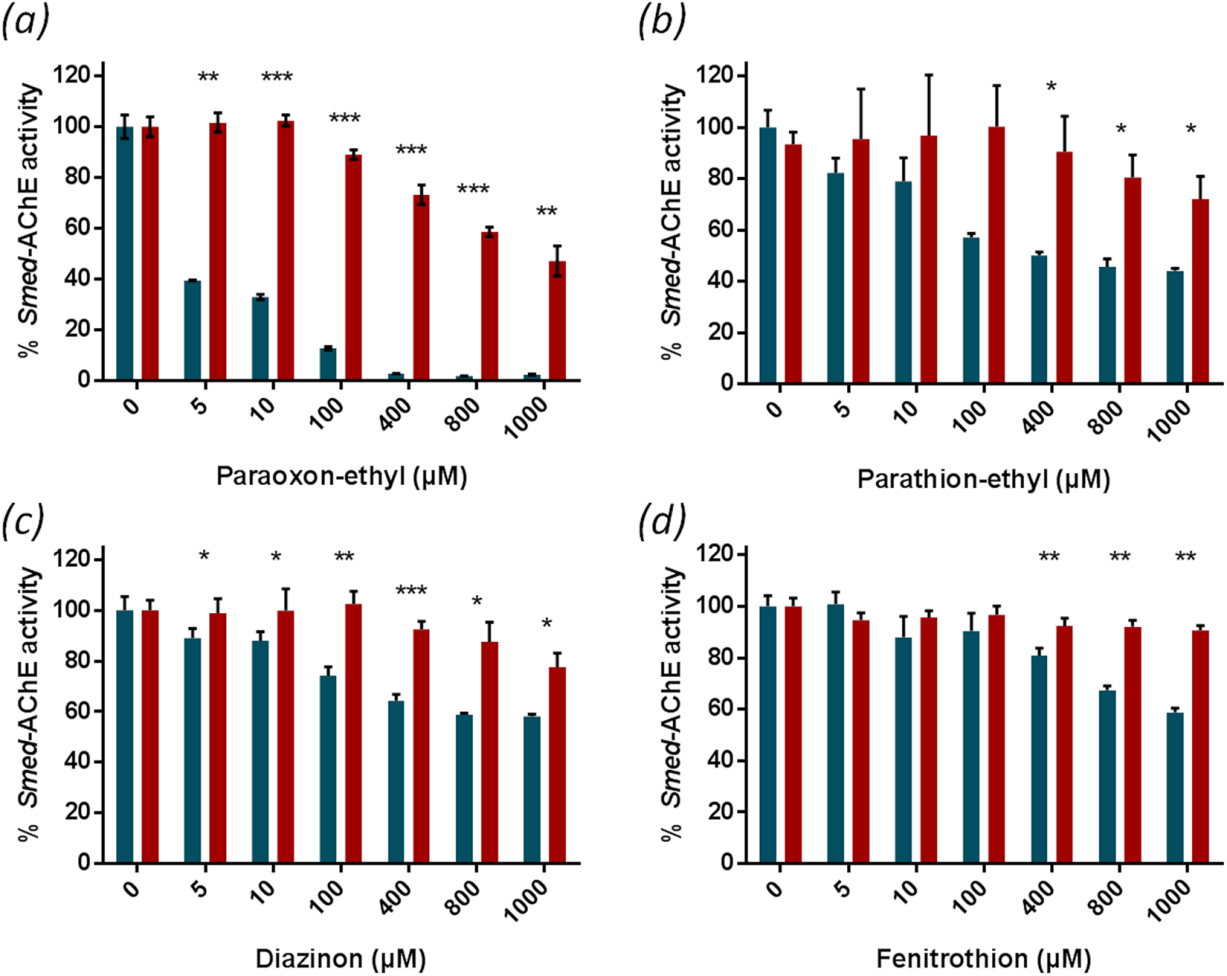
Characterization of Smed-AChE activity in planarian homogenates. Evaluation of *Smed-*AChE inhibition by insecticides and the associated enzymatically-generated degradation products in planarian homogenates. Blue and red bars represent the inhibition of *Smed*-AChE by insecticides and their degradation products respectively for paraoxon-ethyl (**a**), parathion-ethyl (**b**), diazinon (**c**) and fenitrothion (**d**). The values represent the mean ± SD (standard deviation) of three replicates. Black stars (*) indicate a significant difference between insecticide and degradation product for a given concentration (one star to indicate p < 0.05; two stars to indicate p < 0.005; three stars to indicate p < 0.0005 according to Student’s *t*-test).

Acetylcholinesterase activity was determined in *S. mediterranea* using acetylthiocholine as a substrate and Ellman’s assay^36^. Significant activity was measured in planarian homogenates, suggesting the presence of AChE in *S. mediterranea* **(**Fig. 3 **)**. As organophosphorus insecticides are spread in the millimolar range, a panel of concentrations from the micromolar up to the millimolar scale was considered. Acetylcholinesterase activity was reduced when incubated with insecticides. *Smed*-AChE activity was almost zero when paraoxon-ethyl concentration reached 400 µM, while 40 to 60% of activity was retained with 1 mM of parathion-ethyl and diazinon or fenitrothion respectively. Paraoxon-ethyl had a greater impact on *Smed*-AChE activity than parathion-ethyl and other assayed insecticides, suggesting that the oxo form is more toxic than thiono-derivatives. In contrast to insecticides, AChE activity was only slightly affected after enzymatic degradation of the molecules, suggesting that degradation products are poor inhibitors of *Smed*-AChE. Nevertheless, paraoxon-ethyl degradation products were found to decrease *Smed*-AChE activity to 50% at high concentrations.

### Toxicity of OPs in *S. mediterranea*

**Figure 4:**
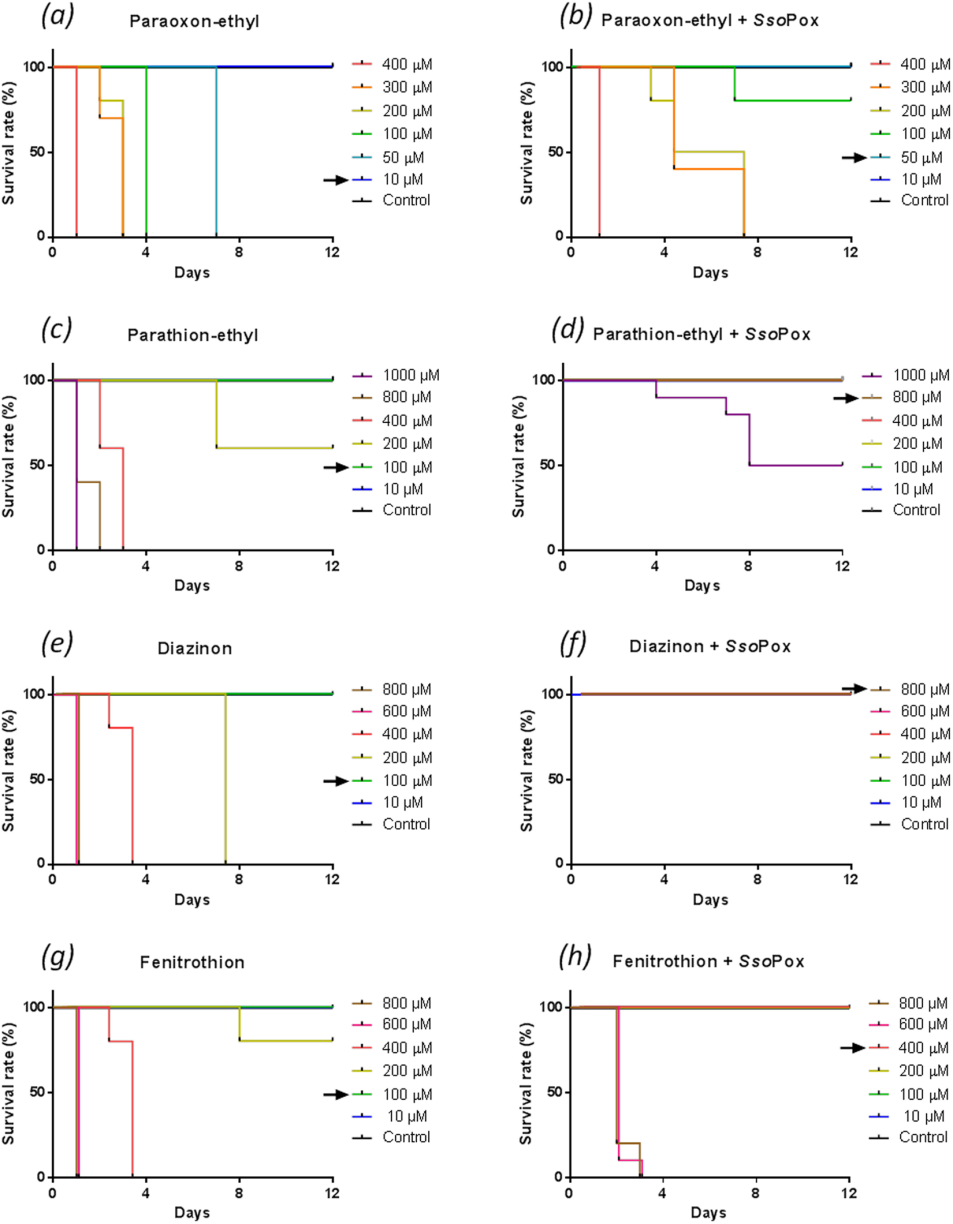
Enzymatic degradation of insecticides increases survival rate in planarians. The toxicity of paraoxon-ethyl, parathion-ethyl, diazinon and fenitrothion before and after degradation by *Sso*Pox was evaluated for concentrations ranging from 10 µM up to 1000 µM. The curves represent the survival rates for 10 planarians exposed to pesticides and the enzymatically generated degradation products paraoxon-ethyl (**a**,**b**), parathion-ethyl (**c**,**d**), diazinon (**e**,**f**), fenitrothion (**g**,**h**). Arrows indicate NOEC values.

The toxicity of four widespread insecticides (paraoxon-ethyl, parathion-ethyl, diazinon, fenitrothion) was evaluated. Dose-responses were assessed by following the survival rate of planarians exposed to increasing concentrations of chemicals (Fig. 4a,c,e,g). Paraoxon-ethyl was entirely fatal down to 50 µM, while no mortality was observed for 10 µM (NOEC for “No Observed Effect Concentration” regarding mortality results) after 12 days. Parathion-ethyl, the thiono-analogue of paraoxon-ethyl was much less toxic than its oxono counterpart, being completely lethal down to 400 µM and with a NOEC value of 100 µM. Diazinon and fenitrothion induced complete mortality at 200 µM and 400 µM and showed NOEC values of 100 µM after 12 days of exposure.

Enzymatic degradation of OPs drastically decreased the deleterious effects in planarians (Fig. 4b,d,f,h, Supplementary Figure 1). After complete hydrolysis, diazinon did not induce any mortality for the tested concentrations. NOEC values for parathion-ethyl and fenitrothion were increased up to 800 µM and 400 µM respectively. The degradation products of parathion-ethyl did not induce complete mortality even for the highest assayed concentration (1 mM). After paraoxon-ethyl degradation, significant toxicity was observed for the enzymatically generated products, albeit at lower levels than for the initial insecticide. NOEC was increased to 50 µM but concentrations from 200 µM up to 400 µM were still fatal to planarians. After enzymatic degradation of OPs, NOEC values were increased by four and five-fold for fenitrothion and paraoxon-ethyl respectively and by up to eight-fold for both parathion-ethyl and diazinon (Supplementary Table 1).

### Effect of OPs on mobility

**Figure 5:**
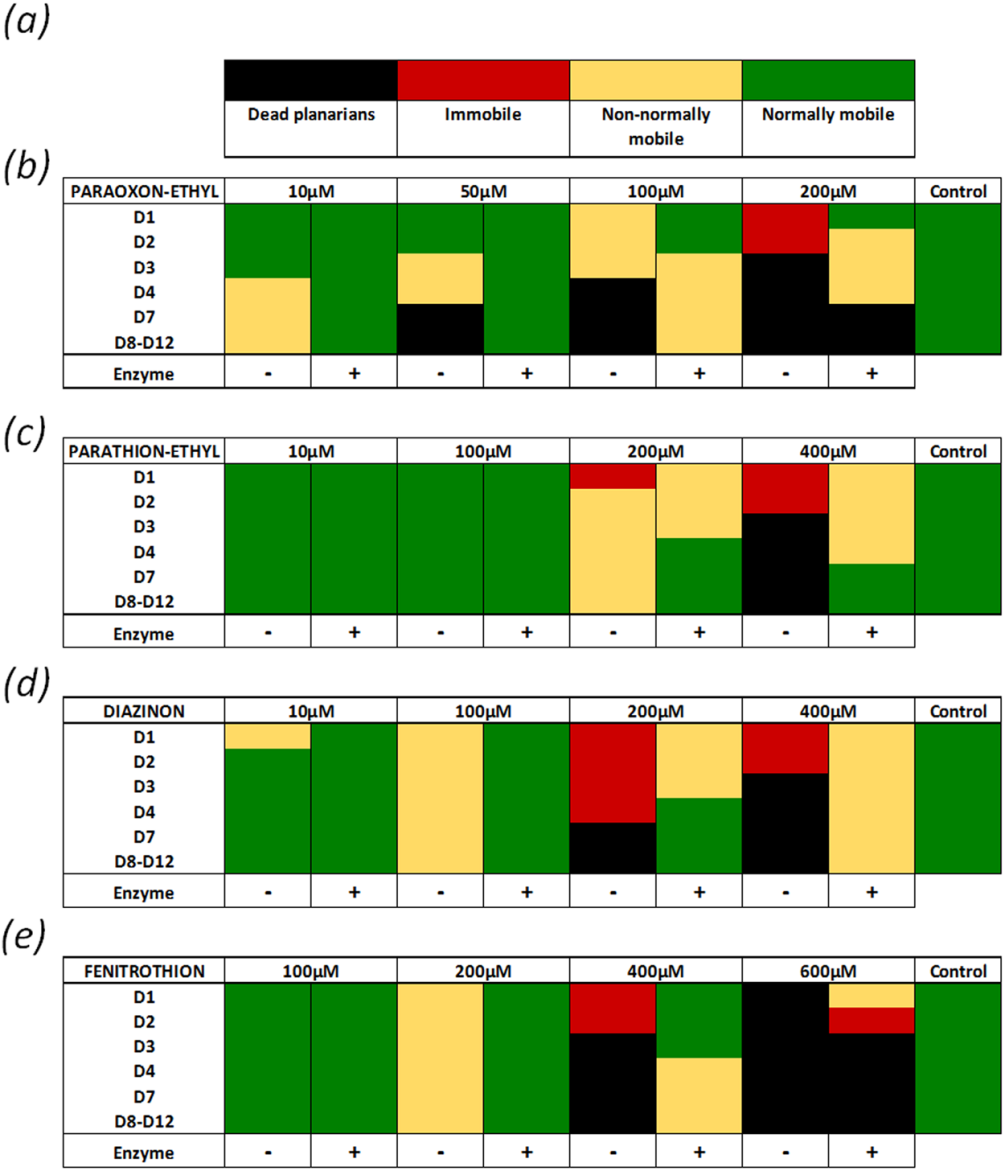
12 days’ incubation of planarians in insecticides and related degradation product solutions and evaluation of the impact on mobility. The average mobility of ten planarians for 12 days (D1 to D12) is presented using a color code describing normal mobility (green), non-normal mobility (yellow), immobility (red) and death (black) (**a**). (+) and (−) describe the solution with and without enzymatic degradation. Paraoxon-ethyl (**b**), parathion-ethyl (**c**), diazinon (**d**), fenitrothion (**e**) and the related degradation products were evaluated. As pesticides were initially solubilized in ethanol before dilution in water to reach final concentration, the control solutions represent the maximum final ethanol concentration for each insecticide.

The impact of insecticides and their associated degradation products on planarian behavior was evaluated (Fig. 5a–e). Planarians were incubated in aqueous solutions containing lethal or sub-lethal concentrations of insecticides. For all insecticides, disruption of mobility was observed. The modifications of mobility were classified into three types: “mobile at normal speed” when planarians moved normally; “immobile” when planarians were prostrate and “non-normally mobile” when planarians were neither normally mobile nor immobile; (Supplementary Figure 2, Supplementary Video 1). Direct visual evaluation of mobility was performed by several observers blinded to the treatment. These observations were reproducible and did not affect the results. The highest concentrations of insecticides led to complete immobility of planarians, followed by death. Low concentrations altered mobility by significantly slowing down the planarian and no or low modification was observed for the lowest 10 µM concentration. After enzymatic degradation of insecticides, planarian mobility was drastically enhanced. Planarians recovered normal behavior for concentrations up to 50 µM, 100 µM and 200 µM for paraoxon-ethyl, diazinon and fenitrothion respectively. For the highest concentrations, enzymatic degradation improved both mobility and survival rate although no complete remission was observed. Mortality was still observed after degradation by the enzyme at concentrations of 200 µM and 600 µM of paraoxon-ethyl and fenitrothion respectively. Ten worms were used for each concentration and the phenotype presented in Fig. 5 was reproducible and was shared by a minimum of 80% of the population. As pesticides were initially prepared in ethanol before dilution in water to reach final concentration, control solutions with the final ethanol concentrations for each condition were performed. We found no effect of ethanol up to the maximum concentration assayed of 0.4%.

### Effect on planarian regeneration

**Figure 6:**
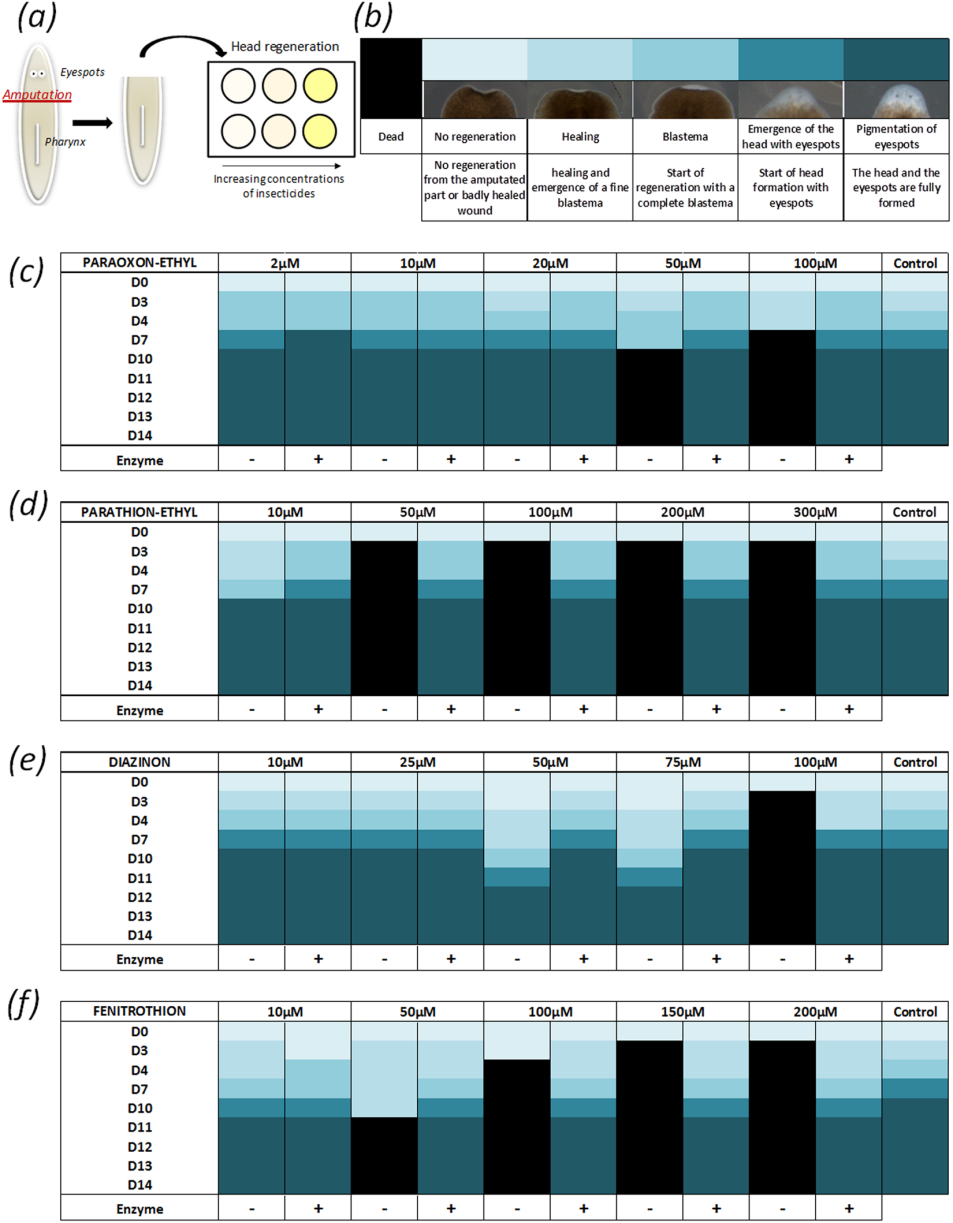
Evaluating the impact of insecticides and their enzymatically-generated degradation products on planarian head regeneration. Five planarians were cut above the pharynx and incubated in insecticide solutions (**a**). Head regeneration of planarians over 15 days (D0 to D14) is presented using a color code from light to dark blue describing initial to final regeneration stages as presented in figure caption (**b**). Paraoxon-ethyl (**c**), parathion-ethyl (**d**), diazinon (**e**), fenitrothion (**f**) and the related degradation products were evaluated. Dead planarians are colored black. (+) and (−) Describe the solution with and without enzymatic degradation. As pesticides were initially solubilized in ethanol before dilution in water to reach final concentration, the control solutions represent the maximum final ethanol concentration for each insecticide.

Planarians have the capacity to regenerate an entire worm from a tissue fragment. Under normal conditions, regeneration is complete 14–15 days after amputation. The effect of sublethal or partially lethal concentration exposure to insecticides and their degradation products, on planarian regeneration was monitored over 15 days (Fig. 6a–f). Head and tail regeneration were followed from tail and head fragments respectively. A visual categorization was used for representing regeneration stages. These criteria were used to characterize the regeneration process and were sufficiently clear to be not dependent on the observer, three different experimentors obtaining the same results.

Insecticides were found to drastically alter head regeneration. Lethal concentrations were decreased down to 100 µM for diazinon and down to 50 µM for parathion-ethyl and fenitrothion as compared to whole planarians. NOEC values were decreased for parathion-ethyl, diazinon and fenitrothion down to 10 µM, 75 µM and 10 µM respectively (Supplementary Table 1). For diazinon and fenitrothion, regeneration progressively improved when the concentration was decreased, while a binary behavior was observed for paraoxon-ethyl and parathion-ethyl at threshold concentration of 50 µM. Impressively, no alteration of the regeneration was observed after the enzymatic decontamination of insecticides. Interestingly, no ectopic growth was observed after one regeneration cycle. Five worms were used for each concentration and the phenotype presented in Fig. 6 was reproducible and was shared by a minimum of 80% of the population. As for mobility and toxicity analyses, no effect of ethanol was observed in the range of the tested concentrations. Very similar results were obtained when studying tail regeneration (Supplementary Figure 3a–f).

## Discussion

The toxicity of four widespread insecticides (paraoxon-ethyl, parathion-ethyl, diazinon, fenitrothion) and their corresponding degradation products generated through enzyme hydrolysis was addressed. The cleavage sites for each substrate were unambiguously confirmed by GC/MS analysis and were found to be in accordance with previously reported catalytic mechanisms^13^. OPs are inhibitors of acetylcholinesterase, but little is known about their toxicity after degradation. To tackle this issue, the planarian, an original flatworm from Platyhelminthes was considered, as this model can be used to evaluate both toxicity and regeneration disruption.

Acetylcholinesterase activity of *S. mediterranea* homogenates was confirmed and this activity was further shown to be susceptible to OP exposure. All four insecticides decreased *Smed*-AChE activity after incubation, although paraoxon-ethyl was the most detrimental substrate. Paraoxon-ethyl had a greater impact on *Smed*-AChE activity than parathion-ethyl, suggesting that, like *Hs*-AChE, *Smed*-AChE is significantly affected by OP in the oxon form. Indeed, many studies have demonstrated that oxon insecticides are drastically more toxic than phosphorothionates which are first activated *in vivo* during metabolization by cytochrome P450 monooxygenase^37–39^. Phylogenetic analysis also showed that predicted AChE from planarians are clustered in a same branch of Platyhelminthes connected to the Chordata phylum, thus confirming that planarians may share an ancient common ancestor with mammalians, as pointed out in a previous study^24^.

Degradation products had a lower impact on *Smed*-AChE activity than insecticides. Degradation of parathion-ethyl, diazinon and fenitrothion led to very low *Smed*-AChE inhibition, underlining their moderate neurotoxicity. Only paraoxon-ethyl degradation products had a significant impact on *Smed*-AChE activity at concentration greater than 400 µM. Because paraoxon-ethyl and parathion-ethyl share one of their two degradation products, namely 4-nitrophenol, it can be safely assumed that the toxic effect observed after paraoxon-ethyl degradation is due to the phosphodiester released, while the phosphorothionate diester analogue, generated through parathion-ethyl degradation, is less toxic. It should be noted that measurements of *Smed*-AChE activity were performed with crude planarian homogenates containing other proteins and metabolites that may also interact with the compounds. Nevertheless, similar inhibitions of AChE were observed when exposing whole planarians in insecticides.

The deleterious effect of OP exposure in whole planarians was further evaluated after incubation in diluted insecticide solution. Insecticide concentrations from 10 µM up to 1 mM were assayed and were in accordance with previous studies. Concentrations up to 800 µM paraoxon-ethyl were used for incubating planarians^25^, while concentrations ranging from 13 µM up to 57 µM were reported for diazinon, fenitrothion, parathion, chlorpyrifos and malathion^26,27^. The viability of *D. japonica* for concentrations up to 500 µM was also described and the authors showed that dichlorvos was 100 times more toxic than chlorpyrifos suggesting that toxicity is strongly dependent on the insecticide^22^. This difference was supposed to be related with the oxon form of dichlorvos which is probably more toxic than thiono insecticides. The toxicity of oxon insecticides was further highlighted in *Djap*-AChE and diazinon oxon was shown to have the highest effect^24^. In this study we found that paraoxon-ethyl was highly toxic in *S. mediterranea*, causing complete mortality at concentrations down to 50 µM. Thiono insecticides were found to be less toxic, causing complete mortality at concentrations down to 200 µM for diazinon and 400 µM for both parathion-ethyl and fenitrothion. After enzymatic degradation of insecticides, the survival of planarians was drastically enhanced. Diazinon and parathion-ethyl have two distinct leaving groups but generate the same phosphorothionate diester. The differences observed in the toxicity of these compounds after decontamination may thus be strongly ascribable to their variable leaving group. Moreover, because only low impacts of the degradation products on *Smed*-AChE activity were observed, their neurotoxicity appears lower as compared to insecticides.

The effect of insecticides on planarian mobility was also evaluated. At sub-lethal concentrations, paraoxon-ethyl and diazinon induced mobility disruption down to 10 µM, while the observable effects of parathion-ethyl and fenitrothion occurred only at 200 µM. At low but lethal concentrations, the mobility of planarians was strongly altered in the early days of exposure and was followed by death. Enzymatic decontamination of insecticides before exposure of planarians enhanced mobility. For most assayed concentrations, a non-exposed type behavior was recovered and enzyme degradation enhanced the survival rate. However, for the highest concentrations of paraoxon-ethyl and fenitrothion, death still occurred but was delayed in comparison to insecticide-treated samples. These results highlight that the enzyme improved planarian mobility, although for highest assayed concentrations, degradation products may also cause disruption, while only a low impact on *Smed*-AChE activity was observed. Although degradation products induced deleterious effects on mobility from 200 µM for 4-nitrophenol and 6-methyl-2-propan-2-γl-1H-pyrimidin-4-one or 400 µM for 3-methyl-4-nitrophenol their impact on planarian behavior was reduced as compared to insecticides.

To further investigate the toxicity of insecticides and their degradation products, their impact on planarian regeneration was observed. Planarians have the capacity to regenerate an entire flatworm from a tissue fragment thanks to abundant stem cells. Alteration of this capacity by low-level OP exposure was followed by a 15 day long full regeneration cycle. The worms were cut into two, allowing both head and tail regeneration from tail and head fragments respectively. Surprisingly, both fragments appeared to be more sensitive to OP exposure than full worms and were characterized by lower NOEC values, as previously discussed. The lowest concentrations did not induce any delay in regeneration, but increasing insecticide concentration either delayed regeneration or caused mortality. In a few cases, planarians died after one week, although the regeneration process had started. Impressively, for all the conditions that were assayed, enzymatic decontamination improved survival and/or regeneration of planarian fragments. No disruption was observed after paraoxon-ethyl, parathion-ethyl and diazinon degradation and only a slight delay was observed for fenitrothion degradation products. Taken together, these results suggest that insecticides may have a stronger impact on planarian development than their degradation products. However, the degradation products were only found to slightly alter *Smed*-AChE activity in contrast to insecticides, underlining a lower neurotoxicity^40^.

Enzymatic remediation of insecticides appears to be a promising approach to decreasing the deleterious effects induced by OP exposure. *Sso*Pox, which is active towards a broad range of OPs and which is highly stable may thus be further considered for decontamination purposes. Previous reports have shown that the enzyme can be produced at a large scale and retain its catalytic activity after immobilization into alginate beads or polyurethanes which may be considered in particular for incorporation into filtration devices^16,17^. Although degradation products may not be completely innocuous, these compounds were found to be much less toxic than insecticides and reduced poisoning effects by increasing NOEC values by up to eight-fold. Enzymatic decontamination also drastically limited the detrimental consequences to planarian mobility and enabled recovery of a non-exposed like regeneration process.

## Materials and Methods

### Production-purification of *Sso*Pox-αsD6

*Sso*Pox-αsD6 was cloned in a pET32b-Δtrx plasmid and produced using an *E. coli* BL21(DE_3_)-pGro7/GroEL (TaKaRa) chaperone expressing strain as previously described^11,12^. In short, auto-inducible ZYP medium (supplemented with 100 µg.ml^−1^ ampicillin and 34 µg.ml^−1^ chloramphenicol) was used for growth. When OD_600nm_ reached a value of 0.8–1, CoCl_2_ and L-arabinose was added (final concentrations respectively 0.2 mM and 2 g.l^−1^) to induce chaperones GroEL/ES expression, the temperature was decreased to 23 °C for 16–20 hours. Cells were harvested by centrifugation (4,400 g, 4 °C, 20 minutes) and the pellet was resuspended in *lysis buffer* (50 mM HEPES pH 8.0, 150 mM NaCl, CoCl_2_ 0.2 mM, lysozyme 0.25 mg.ml^−1^, PMSF 0.1 mM and DNAseI 10 µg.ml^−1^) and stored at −80 °C. The lysate was thawed and lysed by three 30-second steps of sonication (Qsonica, Q700; Amplitude 45). Cell debris were removed by centrifugation (14,000 g, 4 °C, 15 minutes). As *Sso*Pox-αsD6 is hyperthermostable a pre-purification step was conducted by heating the lysate for 30 minutes at 70 °C. Precipitated *E. coli* proteins were removed by centrifugation (14,000 g, 4 °C, 15 minutes). *Sso*Pox-αsD6 was concentrated by ammonium sulfate precipitation (75%) and resuspended in 8 ml of *activity buffer* (50 mM HEPES pH 8.0, 150 mM NaCl, CoCl_2_ 0.2 mM). The remaining ammonium sulfate was eliminated by desalting column (HiPrep 26/10 desalting, GE Healthcare; ÄKTA Avant), concentrated to 2 ml and injected on size exclusion chromatography (HiLoad 16/600 Superdex^TM^ 75 pg, GE Healthcare; ÄKTA Avant). Final purity was verified by 10% SDS-page and protein concentration was determined with a NanoDrop 2000 spectrophotometer (Thermo Scientific).

### Planarian

Freshwater planarians belonging to the *Schmidtea mediterranea* species (asexual clonal line ClW4) were used for all experiments. The planarians were maintained in autoclaved water at 19 °C in the dark and fed twice per week with calf liver. The animals were starved for at least one week prior to the experiments. The water was changed every two days and did not contains antibiotics.

### Acetylcholinesterase activity assays

Acetylcholinesterase activity was measured using Ellman’s assay^36^. Worms were manually selected to fall within a certain range of sizes, around 0.8–1 cm in length. Thirty animals were processed in a Fastprep 24 5 g (three cycles of 10 seconds, 6,0 m/s) in 600 µL of activity buffer (20 mM 2-amino-2-(hydroxymethyl)propane-1,3-diol, 100 mM NaCl, pH 8.0). Experiments were conducted in the same buffer complemented with 4 mM 5,5′-Dithiobis (2-nitrobenzoic acid). 10 µL of the planarian supernatant was added to 140 µL of activity buffer in a 96-well plate. 50 µL of 0.01 M acetylthiocholine iodide in the activity buffer was added to each well to start the reaction and the OD_412nm_ was followed with a microplate reader (Synergy HT, BioTek, USA) for 10 minutes. This activity was normalized to 100%. As negative controls, experiments without planarian supernatant and without acetylthiocholine iodide were carried out. The reproducibility of the crude homogenate preparation was demonstrated with three independent replicates (Supplementary Figure 4).

Experiments with pesticides (paraoxon-ethyl, parathion-ethyl, diazinon and fenitrothion) were conducted as previously described after incubation of the supernatant from the planarian with the pesticide for 45 minutes to ensure reaching inhibition plateau (Supplementary Figure 5). An additional control was carried out using only pesticide in the activity buffer.

Experiments with degradation products of pesticides were carried out as previously described after a degradation by *Sso*Pox-αsD6 variant at a final concentration of 0.25 µM. The reaction was monitored by measuring the absorbance of the product released until complete degradation. The planarians were then incubated for 45 minutes in the decontaminated solution (Supplementary Figure 1). Supplementary controls were conducted using only the degradation products in the activity buffer.

The activity results after incubation with pesticides or degradation products were normalized as compared to the activity value without inhibition set to 100%.

### Toxicity assays and perturbation of behavior

Worms were manually selected to fall within a certain range of sizes, around 0.8–1 cm in length. The animals were starved for at least one week prior to the experiments. Observations were carried out using a Leica M165 FC lens connected to the IC Capture 2.4 software. Four pesticides (Sigma Aldrich) were used for these experiments at different concentrations (paraoxon-ethyl, parathion-ethyl, diazinon and fenitrothion).

4 mL of solution was prepared for each organophosphorus compound at different concentrations (paraoxon-ethyl: 10 µM–400 µM, parathion-ethyl: 10 µM–1000 µM, diazinon: 10 µM–800 µM and fenitrothion: 10 µM–800 µM) without and with degradation by *Sso*Pox-αsD6 variant at a final concentration of 0.25 µM into a 12-well plate. Five planarians were used in each well and experiments were conducted in duplicate. In the same well, mortality and mobility were analyzed over 12 days. As a negative control, the same experiments were carried out without pesticides, with enzyme, and with the pesticide solvent alone (ethanol) at the higher concentration of pesticides.

### Perturbation of development

To study regenerating animals, specimens were cut into two, the head and the tail, using a scalpel just above the pharynx. 4 mL of solution was prepared in a 12-well plate for each organophosphorus compound at different sub-lethal concentrations determined from toxicity assays (paraoxon-ethyl: 2 µM–100 µM, parathion-ethyl: 10 µM–300 µM, diazinon: 10 µM–100 µM and fenitrothion: 10 µM–200 µM) without and with degradation by *Sso*Pox-αsD6 variant at a final concentration of 0.25 µM. After amputation of five planarians, the heads and tails were regrouped respectively in a well and regeneration was monitored over 14 days. As a negative control, the same experiments were conducted without pesticides, with enzyme, and with the pesticide solvent alone (ethanol).

### GC/MS analysis of degradation products

In order to identify the degradation products, 100 µL of the pesticide solution degraded by *Sso*Pox-αsD6 variant was first extracted with 100 µL of chloroform. Organic extracts were analyzed using a Clarus 500 gas chromatograph equipped with a SQ8S MS detector (Perkin Elmer, Courtaboeuf, France). 1 µL of organic extract was volatilized at 220 °C (split 15 mL.min^−1^) in a deactivated FocusLiner with quartz wool (SGE, Ringwood, Australia) and compounds were separated on an Elite-5MS column (30 m, 0.25 mm i.d., 0.25 mm film thickness) for 12 minutes using a temperature gradient (80–280 °C at 30 °C.min^−1^, five minutes’ hold). Helium flowing at 2 mL.min^−1^ was used as carrier gas. The MS inlet line was set at 280 °C and electron ionization source at 280 °C and 70 eV. Full scan monitoring was performed from 40 to 400 *m*/*z* to identify chemicals by spectral database search using MS Search 2.0 operated with the Standard Reference Database 1 A (National Institute of Standards and Technology, Gaithersburg, MD, USA). Identification was confirmed with reverse and forward scores above 800. Product identities were then validated with the Kovats Retention Index from the injection of an alkane standard (Sigma Aldrich, France). In the case of weak degradation product signals (4-nitrophenol *m/z* 139, 6-methyl-2-propan-2-yl-1H-pyrimidin-4-one *m/z* 137, 3-methyl-4-nitrophenol *m*/*z* 136), extracted ion chromatograms were generated with base peak ions to confirm the absence of chemicals (Paraoxon-ethyl and parathion-ethyl *m/z* 109; Fenitrothion *m/z* 125; Diazinon *m/z* 179). All data were processed using Turbomass 6.1 (Perkin Elmer).

### Phylogeny

To identify a planarian homolog of *Homo sapiens* AChE (*Hs-*AChE) isoform E4-E5 precursor (NP_056646.1), we examined, via TBLASTN, the planarian transcriptome database PlanMine (http://planmine.mpi-cbg.de/planmine/begin.do)^33^ particularly the following flatworm species: *S. mediterranea, P. torva, P. tenuis, D. lactum* and *S. polychroa*. We identified sequences producing a significant alignment with the genes of interest. The top BLAST hit was used to predict *Smed-*AChE, *Pto-*AChE, *Pte-*AChE, *Dl-*AChE and *Sp-*AChE via FGENESH+ (http://www.softberry.com/). Homology at the protein level between predicted *Smed-*AChE and *Hs-*AChE was analyzed using BLAST.

The previous predicted sequences were used as template for BLAST in UNIPROT (http://www.uniprot.org/blast/) and 250 sequences were collected. Sequence alignment, including planarian predicted sequences and Phylogeny, were performed with MEGA^34,35^. A maximum likelihood tree was constructed and tested using the bootstrap method with a 100 replications.

### Statistical analysis

For *Smed-*AChE activity (Fig. 3) the values represent the mean ± SD (standard deviation) of the percentage of three technical replicates. Black stars (*) indicate a significant difference between insecticide and degradation product for a given concentration. According to Student’s t-test, one star indicates p < 0.05, two stars indicate p < 0.005 and three stars indicate p < 0.0005.

## Electronic supplementary material

Supplementary Info

Supplementary Video 1

## References

[1] Gupta, R. C. *Handbook of Toxicology of Chemical Warfare Agents*. (Academic Press, 2009).

[2] Jaipieam S (2009). Organophosphate Pesticide Residues in Drinking Water from Artesian Wells and Health Risk Assessment of Agricultural Communities, Thailand. Hum. Ecol. Risk Assess. Int. J..

[3] Beauregard G, Lum J, Roufogalis BD (1981). Effect of histidine modification on the aging of organophosphate-inhibited acetylcholinesterase. Biochem. Pharmacol..

[4] Zwiener RJ, Ginsburg CM (1988). Organophosphate and Carbamate Poisoning in Infants and Children. Pediatrics.

[5] Lessenger JE, Reese BE (1999). Rational Use of Cholinesterase Activity Testing in Pesticide Poisoning. J. Am. Board Fam. Pract..

[6] De Silva HJ, Samarawickrema NA, Wickremasinghe AR (2006). Toxicity due to organophosphorus compounds: what about chronic exposure?. Trans. R. Soc. Trop. Med. Hyg..

[7] Ray DE, Richards PG (2001). The potential for toxic effects of chronic, low-dose exposure to organophosphates. Toxicol. Lett..

[8] Maxwell DM, Brecht KM, Koplovitz I, Sweeney RE (2006). Acetylcholinesterase inhibition: does it explain the toxicity of organophosphorus compounds?. Arch. Toxicol..

[9] Costa LG (2006). Current issues in organophosphate toxicology. Clin. Chim. Acta.

[10] Jacquet, P. *et al*. Current and emerging strategies for organophosphate decontamination: special focus on hyperstable enzymes. *Environ. Sci. Pollut. Res*. 1–19, 10.1007/s11356-016-6143-1 (2016). 10.1007/s11356-016-6143-1 26832878

[11] Hiblot, J., Gotthard, G., Chabriere, E. & Elias, M. Characterisation of the organophosphate hydrolase catalytic activity of SsoPox. *Sci. Rep*. **2**, (2012). 10.1038/srep00779 PMC3493116 23139857

[12] Hiblot J, Gotthard G, Elias M, Chabriere E (2013). Differential Active Site Loop Conformations Mediate Promiscuous Activities in the Lactonase SsoPox. PLoS ONE.

[13] Elias M (2008). Structural Basis for Natural Lactonase and Promiscuous Phosphotriesterase Activities. J. Mol. Biol..

[14] Del Giudice I (2016). An efficient thermostable organophosphate hydrolase and its application in pesticide decontamination. Biotechnol. Bioeng..

[15] Merone L, Mandrich L, Rossi M, Manco G (2005). A thermostable phosphotriesterase from the archaeon Sulfolobus solfataricus: cloning, overexpression and properties. Extremophiles.

[16] Rémy, B. *et al*. Harnessing hyperthermostable lactonase from Sulfolobus solfataricus for biotechnological applications. *Sci. Rep*. **6** (2016). 10.1038/srep37780 PMC5120315 27876889

[17] Guendouze, A. *et al*. Effect of quorum quenching lactonase in clinical isolates of Pseudomonas aeruginosa and comparison with quorum sensing inhibitors. *Front. Microbiol*. **8** (2017). 10.3389/fmicb.2017.00227 PMC5306132 28261183

[18] Vitola G (2016). Polymeric biocatalytic membranes with immobilized thermostable phosphotriesterase. J. Membr. Sci..

[19] Singh BK, Walker A (2006). Microbial degradation of organophosphorus compounds. FEMS Microbiol. Rev..

[20] Conti, F., Abnave, P. & Ghigo, E. Unconventional animal models: a booster for new advances in host—pathogen interactions. *Front. Cell. Infect. Microbiol*. **4** (2014). 10.3389/fcimb.2014.00142 PMC4189411 25340043

[21] Torre C, Ghigo É (2015). Planaria: an immortal worm to clarify human immune response. Médecine Sci. MS.

[22] Hagstrom D, Cochet-Escartin O, Zhang S, Khuu C, Collins E-MS (2015). Freshwater Planarians as an Alternative Animal Model for Neurotoxicology. Toxicol. Sci..

[23] Hagstrom D, Cochet-Escartin O, Collins E-MS (2016). Planarian brain regeneration as a model system for developmental neurotoxicology. Regeneration.

[24] Hagstrom, D. *et al*. Planarian cholinesterase: *in vitro* characterization of an evolutionarily ancient enzyme to study organophosphorus pesticide toxicity and reactivation. *Arch. Toxicol*. 1–11, 10.1007/s00204-016-1908-3 (2016). 10.1007/s00204-016-1908-3 PMC6485937 27990564

[25] Rodríguez HH (2010). The effect of paraoxon on spermatogenesis in Dugesia gonocephala from the Chilean Altiplano: proliferation and apoptosis. Environ. Sci. Pollut. Res..

[26] Villar D, Li MH, Schaeffer DJ (1993). Toxicity of organophosphorus pesticides to Dugesia dorotocephala. Bull. Environ. Contam. Toxicol..

[27] Villar D, González M, Gualda MJ, Schaeffer DJ (1994). Effects of organophosphorus insecticides on Dugesia tigrina: Cholinesterase activity and head regeneration. Bull. Environ. Contam. Toxicol..

[28] Pezzementi L, Nachon F, Chatonnet A (2011). Evolution of Acetylcholinesterase and Butyrylcholinesterase in the Vertebrates: An Atypical Butyrylcholinesterase from the Medaka Oryzias latipes. PLOS ONE.

[29] Pezzementi L, Chatonnet A (2010). Evolution of cholinesterases in the animal kingdom. Chem. Biol. Interact..

[30] Sanders M (1996). Biochemical and molecular characterization of acetylcholinesterase from the hagfish Myxine glutinosa. Comp. Biochem. Physiol. B Biochem. Mol. Biol..

[31] Chatonnet A, Lockridge O (1989). Comparison of butyrylcholinesterase and acetylcholinesterase. Biochem. J..

[32] Reddien PW, Newmark PA, Sánchez Alvarado A (2008). Gene nomenclature guidelines for the planarian Schmidtea mediterranea. Dev. Dyn. Off. Publ. Am. Assoc. Anat..

[33] Grudniewska M (2016). Transcriptional signatures of somatic neoblasts and germline cells in Macrostomum lignano. eLife.

[34] Jones DT, Taylor WR, Thornton JM (1992). The rapid generation of mutation data matrices from protein sequences. Bioinformatics.

[35] Kumar S, Stecher G, Tamura K (2016). MEGA7: Molecular Evolutionary Genetics Analysis Version 7.0 for Bigger Datasets. Mol. Biol. Evol..

[36] Ellman GL, Courtney KD, Andres V, Featherstone RM (1961). A new and rapid colorimetric determination of acetylcholinesterase activity. Biochem. Pharmacol..

[37] Fukuto TR (1990). Mechanism of action of organophosphorus and carbamate insecticides. Environ. Health Perspect..

[38] Mutch E, Blain PG, Williams FM (1999). The role of metabolism in determining susceptibility to parathion toxicity in man. Toxicol. Lett..

[39] Jokanović M (2001). Biotransformation of organophosphorus compounds. Toxicology.

[40] Nishimura K, Kitamura Y, Taniguchi T, Agata K (2010). Analysis of motor function modulated by cholinergic neurons in planarian Dugesia japonica. Neuroscience.

