# Supplementary material for: Enzymatic degradation of organophosphorus insecticides decreases toxicity in planarians and enhances survival
Source: Sci Rep. 2017 Nov 9;7:15194. doi: 10.1038/s41598-017-15209-8 (PMC5680213; doi:10.1038/s41598-017-15209-8)
Supplement: Supplementary file 1 — Supplementary Info [file 41598_2017_15209_MOESM1_ESM.pdf]

Supplementary information

Enzymatic degradation of organophosphorus insecticides decreases toxicity in planarians and enhances survival

Laetitia Poirier[1], Lucile Brun[1], Pauline Jacquet[1], Catherine Lepolard[1], Nicholas Armstrong[1], Cédric Torre[1], David Daudé[2], Eric Ghigo[1] & Eric Chabrière[1]

[1]CNRS UMR 7278, IRD198, INSERM U1095, APHM, Institut Hospitalier Universitaire Méditerranée-Infection, Aix-Marseille Université, 19-21 Bd Jean Moulin 13005 Marseille Cedex 05, France.

[2]Gene&GreenTK, 19-21 Bd Jean Moulin 13005 Marseille Cedex 05, France.

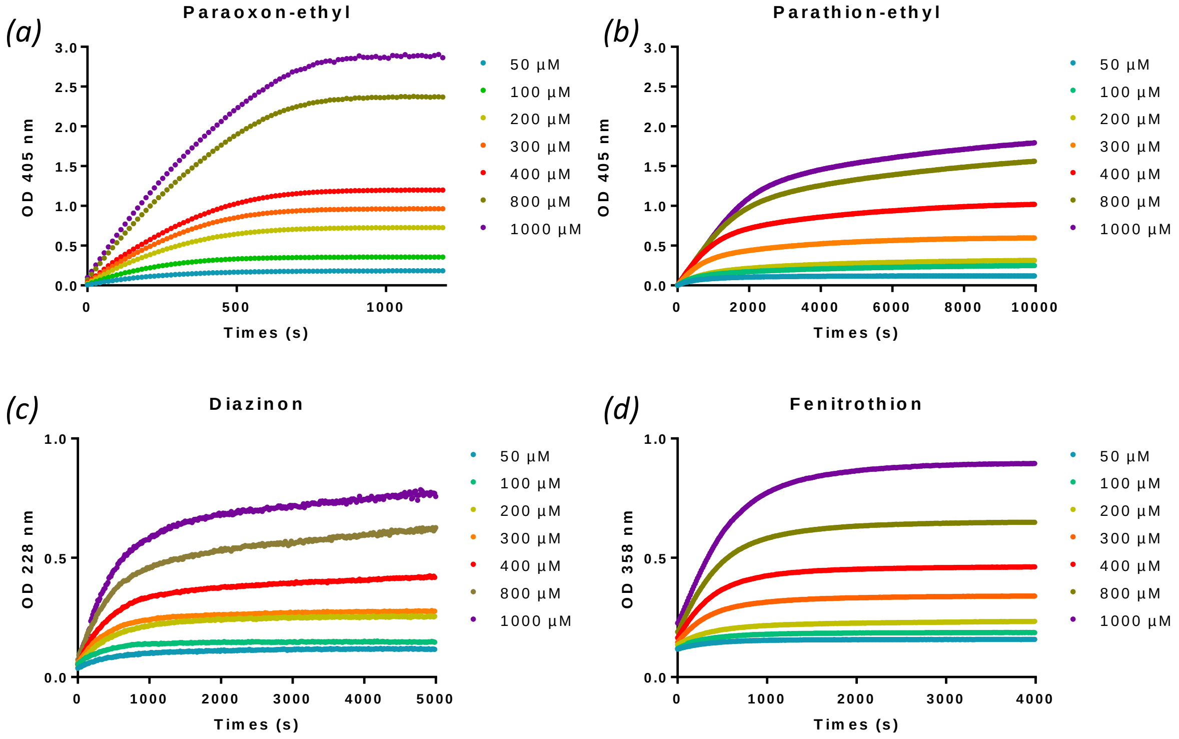

Supplementary figure 1. Enzymatic degradation of insecticides. Insecticide hydrolysis was followed by measuring absorbance of the product released at 405 nm for paraoxon-ethyl (***a***) and parathion-ethyl (***b***), 228 nm for diazinon (***c***) and 358 nm for fenitrothion (***d***). Curves represent the mean of three replicates.

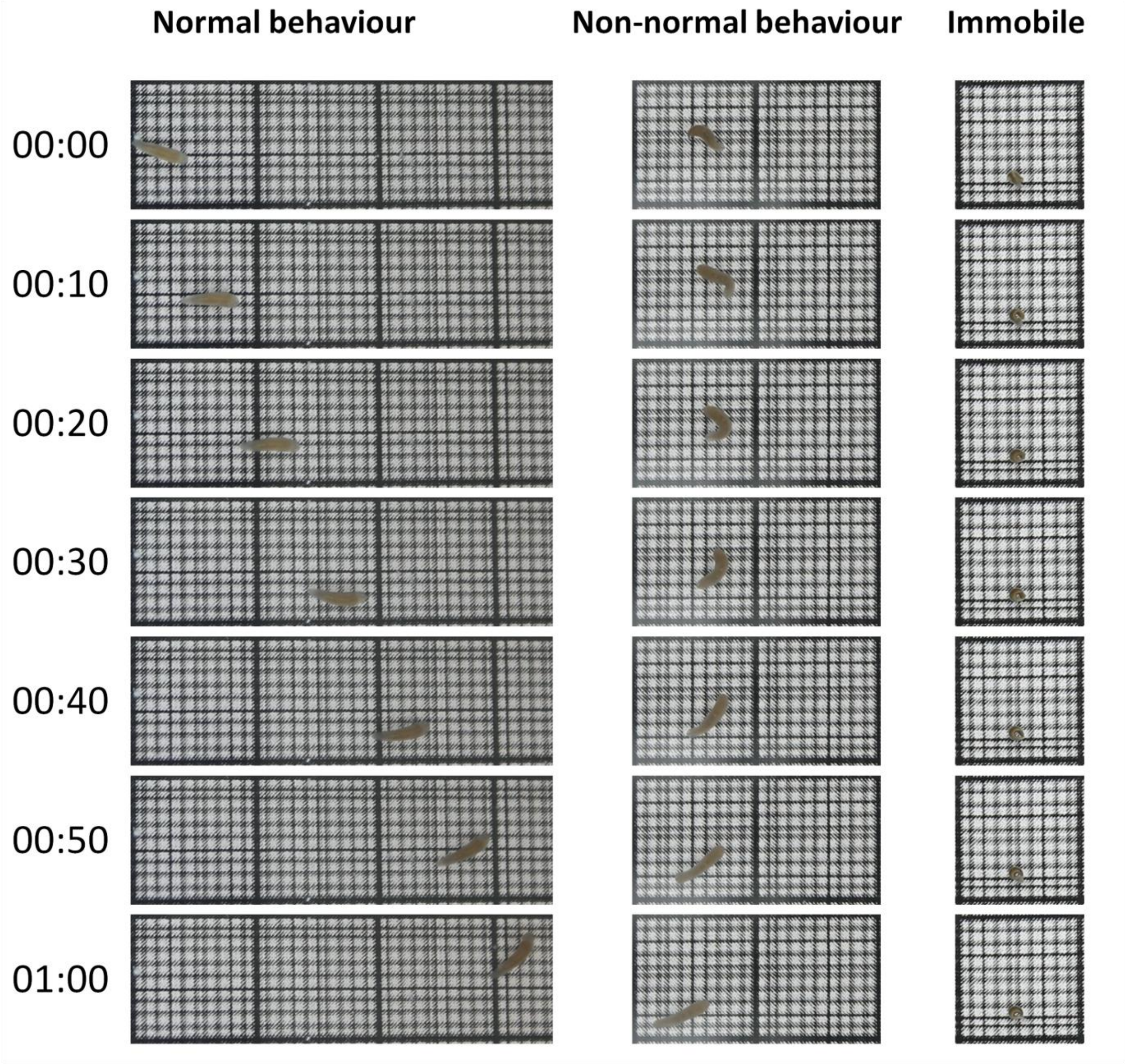

Supplementary figure 2. Defining mobility criteria. The modifications of planarian mobility by insecticides or their degradation products were observed and classified in three categories.

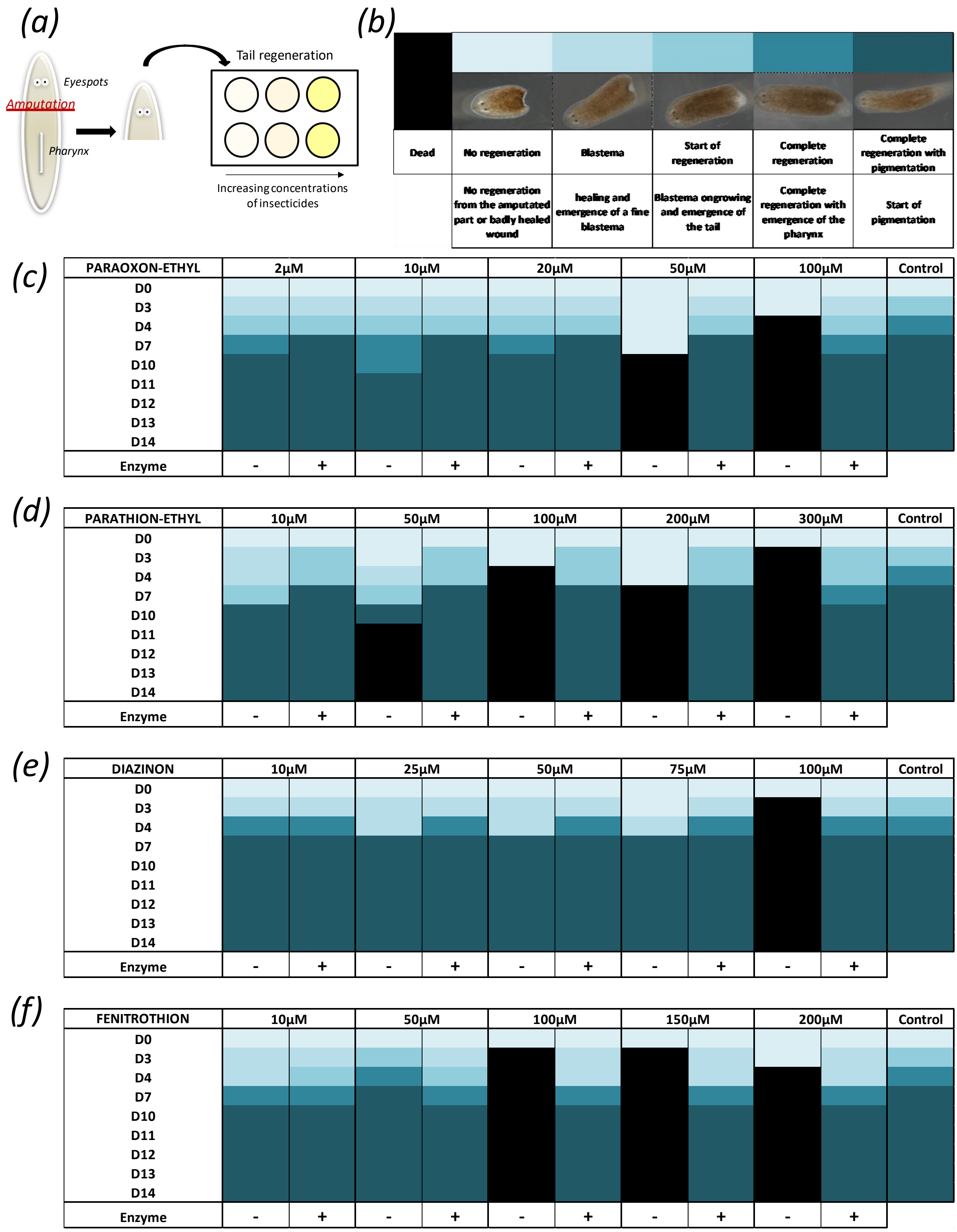

Supplementary figure 3. Evaluating the impact of insecticides and their enzymatically-generated degradation products on planarian tail regeneration. Five planarians were cut above the pharynx and incubated in insecticide solutions (***a***).The tail regeneration of planarians is presented using a color code from light to dark blue describing initial to final regeneration stages as presented in figure caption (***b***). Paraoxon-ethyl (***c***), parathion-ethyl (***d***), diazinon (***e***), fenitrothion (***f***) and the related degradation products were evaluated. Dead planarians are colored black. (+) and (-) describe the solution with and without enzymatic degradation. As

pesticides were initially solubilized in ethanol before dilution in water to reach final concentration, the control solutions represent the maximum final ethanol concentration for each insecticide.

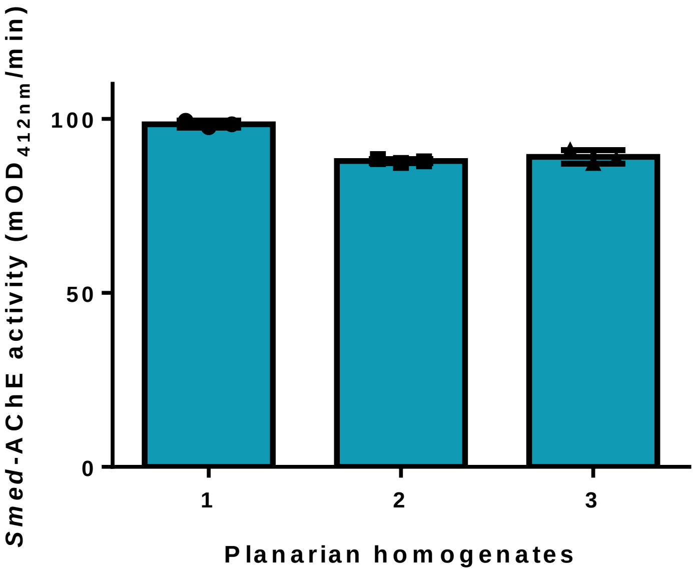

Supplementary figure 4. Evaluating the reproducibility of planarian homogenate preparation. The overall AChE activity of planarians homogenates was consistent for three independent preparations.

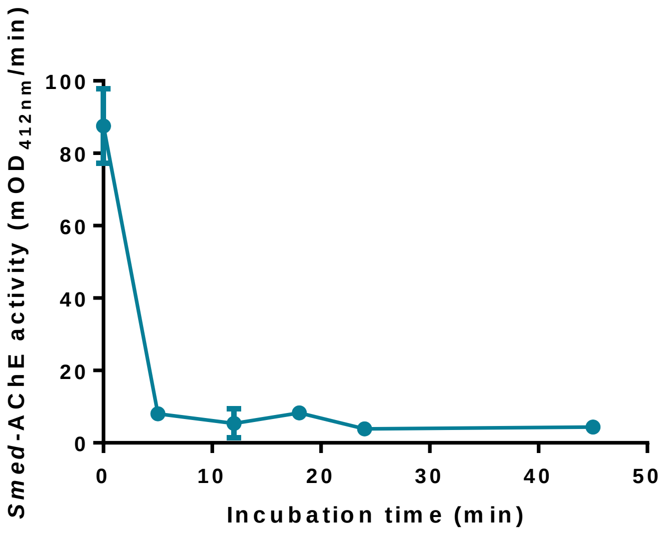

Supplementary figure 5. Influence of incubation time on acetylcholinesterase inhibition with 800 µM paraoxon-ethyl.

Supplementary Table 1. Summary of NOEC values for full and amputated worms incubated in insecticides or degradation products

NOEC	Full worm		Amputated worm			
			Head		Tail	
	Pesticide	Degradation product	Pesticide	Degradation product	Pesticide	Degradation product
Paraoxon-ethyl	10µM	50µM	20µM	≥100µM	20µM	≥100µM
Parathion-ethyl	100µM	800µM	10µM	≥300µM	10µM	≥300µM
Diazinon	100µM	800µM	75µM	≥100µM	75µM	≥100µM
Fenitrothion	100µM	400µM	10µM	≥200µM	50µM	≥200µM
