# Supplementary figures and images for: Enzymatic degradation of organophosphorus insecticides decreases toxicity in planarians and enhances survival
Source: Sci Rep. 2017 Nov 9;7:15194. doi: 10.1038/s41598-017-15209-8 (PMC5680213; doi:10.1038/s41598-017-15209-8)

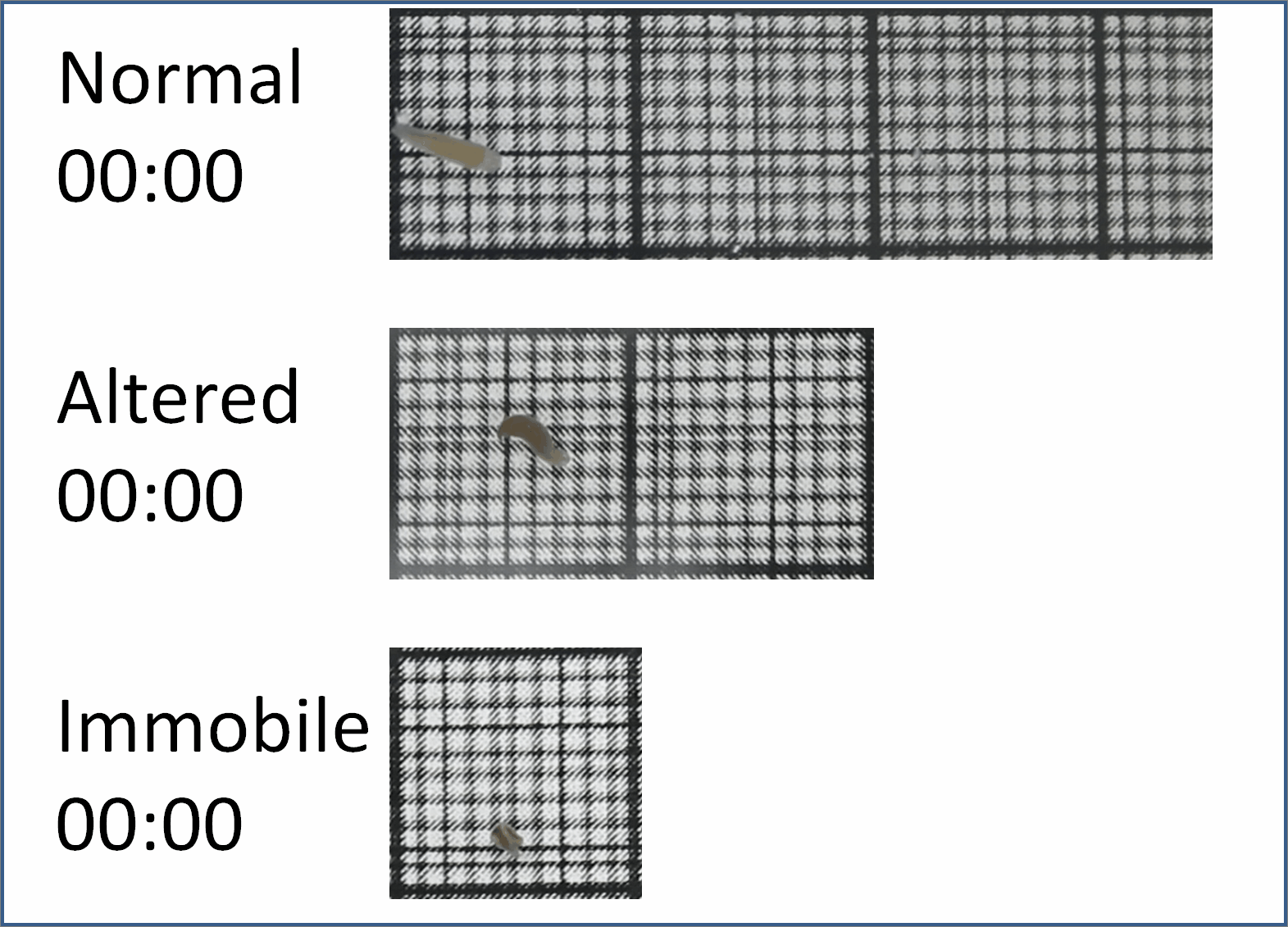

Supplement: Supplementary file 2 — Supplementary Video 1 [file 41598_2017_15209_MOESM2_ESM.gif]
